# Impact of device sales representatives in the operating room on clinical outcomes during standard endovascular aneurysm repair: A retrospective case-control study

**DOI:** 10.1371/journal.pone.0355214

**Published:** 2026-08-04

**Authors:** Farhad Udwadia, Annudesh Liyanage, Gary K. Yang

**Affiliations:** 1 Division of Vascular Surgery, University of British Columbia, Vancouver, British Columbia, Canada; 2 Faculty of Medicine, University of British Columbia, Vancouver, British Columbia, Canada; 3 Division of Vascular Surgery, Kelowna General Hospital, Kelowna, British Columbia, Canada; Ningbo China Institute for Supply Chain Innovation, UNITED STATES OF AMERICA

## Abstract

Device sales representatives (DSRs) play a key role in providing vascular surgeons with device-specific education, operational guidance, and troubleshooting support. However, industry relationships may introduce bias, raising ethical concerns. This study aimed to evaluate the clinical benefit of DSR presence in the operating room (OR) during standard, infrarenal endovascular aortic repair (EVAR). A retrospective, case-control study was conducted to assess the association between DSR presence and surgical outcomes in elective, infrarenal EVAR cases at an experienced endovascular institution. Preoperative (age, gender, ASA classification, and past medical history), perioperative (device company, OR and fluoroscopy time, and use of additional endografts or stents) and postoperative (length of hospital stay post-operation, endoleaks at six weeks and six months, and mortality at six months) variables were collected for cases between June 1st, 2019 to December 31st, 2022. Fisher’s exact test, chi-squared test and student’s t-test were used to compare DSR-present and control groups. Ninety standard infrarenal EVARs were included with a DSR present in 64 (71.1%) cases. Cook and Gore devices were used in roughly equal proportions with a single case using a Medtronic device. DSR presence was not associated with a statistically significant change in operative duration (p = 0.53) or fluoroscopy time (p = 0.95), or in rates of endograft usage (p = 0.35) or adjunctive stent use (p = 1.00). Length of hospital stay (p = 0.46), short- (p = 0.63) and long-term (p = 1.00) endoleaks, and mortality rates (p = 1.00) were similar between groups. In this study of standard, infrarenal EVAR procedures at a high volume endovascular centre, DSR presence was not associated with statistically significant differences in case efficiency, device utilization or clinical outcomes. These findings suggest that routine DSR involvement in such procedures may not provide a clear clinical advantage. In light of important ethical considerations, the study calls for a more selective use of DSR support and for larger, multicentre studies to better understand their role in EVAR cases.

## Introduction

Endovascular approaches have become ubiquitous in vascular surgery with endografts used in several procedures to treat a wide variety of vascular pathology [1]. For infrarenal aortic aneurysms that meet anatomic criteria, endovascular aortic repair (EVAR) has become an accepted mainstay therapy for most patients. With new innovations and increasing user familiarity, endovascular approaches will continue to grow in its applications [2]. Vascular surgeons often work closely with industry partners to enhance device understanding and develop new products. However, this collaboration can result in unintended ethical concerns, such as biasing surgeons towards company products. Additionally, issues related to patient privacy may arise, since device sales representatives (DSRs) are not members of the direct care team and their presence in operating rooms (ORs) is not always disclosed [3].

Vascular surgeons and residents interact routinely with DSRs, who play a role in education and device familiarization. Residents often participate in simulation sessions led by DSRs to learn proper device sizing and gain relevant product knowledge. DSRs also demonstrate new products to surgeons to keep them updated on the latest advancements [4]. For clinical practice, DSRs are consulted in many endovascular cases. They provide their product expertise in planning for these procedures and are often present in the OR to guide device deployment and to troubleshoot malfunctions [5].

In other medical fields, particularly with the pharmaceutical industry, there is evidence of sales representatives influencing physician behavior for profit [6]. Within vascular surgery, our previous survey of Canadian Society for Vascular Surgery members found that most surgeons did not perceive significant ethical issues with DSR presence in the OR. However, many believed their colleagues might be influenced by such collaborations [7]. This raises the question of whether the clinical benefits of DSR presence in the OR outweigh potential ethical concerns.

While our previous work revealed that Canadian vascular surgeons highly value DSRs for case efficiency, planning, and device-specific expertise, there is currently a lack of research examining the direct clinical impact of these relationships [7]. Accordingly, this study aims to evaluate the effect of DSR presence on surgical outcomes, focusing specifically on infrarenal EVAR cases.

## Materials and methods

### Study design

We conducted a retrospective, case-control study to evaluate the impact of DSR presence on surgical outcomes. Institutional approval was obtained from the University of British Columbia Research Ethics Board (H23-02117). Data was accessed on May 10, 2024 for research purposes. Only de-identified data was used during the process of data collection and analysis. The study population included all patients between January 1, 2019, and December 31, 2022, at Kelowna General Hospital, Kelowna, BC, Canada: an experienced endovascular centre. Only elective cases were included, as it is uncommon for DSRs to be present in emergent cases. Additionally, only infrarenal EVAR cases were included to ensure homogeneity in the study population.

### Data collection

Data was extracted from electronic medical records and included basic patient information such as age, gender, the American Society of Anesthesiologists (ASA) physical status classification [8], and relevant past medical history. Procedural data included DSR presence during the operation, device manufacturer, operative and fluoroscopy durations, and the use of additional endografts or stents defined as cases using more than a single endograft or stent. Clinical outcomes were comprised of the length of hospital stay, presence of endoleaks of any type at six weeks and six months post-operation, and mortality at six months. The data collection was performed independently by two researchers (FU, GY), and all findings were entered into a standardized data collection form (S1 File).

### Analysis

For statistical analysis, the data was split into a DSR cohort (with DSR present during the operation) and a control cohort (no DSR present during the operation). SPSS (IMG, Armonk, NY) software was used to identify variables with statistically significant differences. Fisher’s exact test for employed for binary variables, student’s t-test for continuous variables, and chi-squared test for categorical variables with more than two classes. For all tests, statistical significance was assumed with a p-value less than 0.05.

## Results

A total of 90 standard, infrarenal EVARs were included during the three year period and of these procedures, 64 (71.1%) were carried out with a DSR present. Cases were determined to be of similar complexity with standard, infrarenal aneurysm anatomy when screened by the two researchers. The decision to have a DSR present was decided by surgeon preference alone. Baseline patient demographics were similar between DSR and control groups (Table 1).

**Table 1:** Baseline demographics of DSR present and control infrarenal EVAR cases.

	Missing	All	DSR	Control	P-value
n		90	64	26	
**Gender (male)**	0	83 (92.2)	59 (92.2)	24 (92.3)	1.000
**Age (years)**	0	77.2 (7.7)	77.1 (8.1)	77.4 (6.8)	0.863
**ASA Class**	0				0.543
**2**		2 (2.2)	1 (1.6)	1 (3.8)	
**3**		29 (32.2)	19 (29.7)	10 (38.5)	
**4**		59 (65.6)	44 (68.8)	15 (57.7)	
**HTN**	1	53 (58.9)	36 (56.2)	17 (65.4)	0.174
**CAD**	1	29 (32.2)	21 (32.8)	8 (30.8)	0.287
**Arrhythmia**	1	18 (20.0)	12 (18.8)	6 (23.1)	0.248
**COPD**	1	17 (18.9)	14 (21.9)	3 (11.5)	0.441
**OSA**	1	13 (14.4)	9 (14.1)	4 (15.4)	0.280
**Liver Disease**	1	5 (5.6)	4 (6.2)	1 (3.8)	0.265
**CKD**	1	4 (4.4)	4 (6.2)	0 (0.0)	0.129
**DM**	1	18 (20.0)	13 (20.3)	5 (19.2)	0.288
**Stroke/TIA**	1	4 (4.4)	4 (6.2)	0 (0.0)	0.129
**Smoking Status**	16				0.806
**Current**		10 (11.1)	8 (12.5)	2 (7.7)	
**Ex-Smoker**		57 (63.3)	41 (64.1)	16 (61.5)	
**Non-Smoker**		7 (7.8)	5 (7.8)	2 (7.7)	

Continuous variables are reported as [mean + /- one standard deviation] and categorical variables as [number (frequency)]. ASA = American Society of Anesthesiologists classification; HTN = hypertension; CAD = coronary artery disease; COPD = chronic obstructive pulmonary disease; OSA = obstructive sleep apnea; DM = diabetes mellitus; TIA = transient ischemic attack.

Intraprocedural details and clinical outcomes are shown in Table 2. Both groups predominantly utilized Cook and Gore endografts with similar frequencies. There was a single instance of a Medtronic device used within the DSR cohort. The mean operative time was longer in the DSR group by approximately five minutes; however, this difference was not statistically significant (p = 0.53). Similarly, fluoroscopy time did not differ greatly between groups (p = 0.95). The usage rate of additional endografts was lower in the DSR cohort, although this difference did not reach statistical significance (p = 0.35). The usage rate of additional stents was essentially identical across both groups (p = 1.00).

**Table 2:** Intraprocedural details and clinical outcomes in DSR present and control infrarenal EVAR cases.

	Missing	All	DSR	Control	P-value
Device Company					0.797
**Cook**	0	43 (47.8)	30 (46.9)	13 (50.0)	
**Gore**	0	46 (51.1)	33 (51.6)	13 (50.0)	
**Medtronic**	0	1 (1.1)	1 (1.6)	0 (0.0)	
**Surgical Time (min)**	0	74.3 (33.8)	75.5 (37.4)	71.4 (22.9)	0.528
**Fluoroscopy Time (min)**	1	15.6 (9.7)	15.7 (10.4)	15.5 (8.3)	0.945
**Additional Endograft Use**	0	6 (6.7)	3 (4.7)	3 (11.5)	0.350
**Additional Stent Use**	0	4 (4.4)	3 (4.7)	1 (3.8)	1.000
**Length of Stay (days)**	2	2.6 (6.0)	2.2 (2.1)	3.8 (10.7)	0.455
**6-week Endoleak**	2	31 (34.4)	21 (22.8)	10 (38.5)	0.630
**6-month Endoleak**	5	8 (8.9)	6 (9.4)	2 (7.7)	1.000
**6-month Mortality**	5	0 (0.0)	0 (0.0)	0 (0.0)	1.000

Continuous variables are reported as [mean + /- one standard deviation] and categorical variables as [number (frequency)].

Regarding postoperative outcomes, although not statistically significant, the length of hospital stay was on average 1.6 days shorter in the DSR group (p = 0.46) and the incidence of endoleaks at six weeks post-operation was 1.7 times lower in the DSR cohort (p = 0.63). Rates of endoleak and mortality at six months post-operation were comparable between the two groups (p = 1.00 for both).

## Discussion

This study assessed the correlation between DSR presence and surgical outcomes in elective, infrarenal EVAR procedures performed at an experienced endovascular centre. While previous studies have investigated the role of DSRs in the OR for other surgical specialties, its impact in EVAR remains unclear. By selecting a homogeneous, standard-risk EVAR population, this study better contributes procedure-specific results to assess when DSR presence is warranted.

With respect to case efficiency, DSR presence was associated with an average increase in operative time of roughly five minutes, although this was not statistically significant. This differs from results in other surgical fields in which DSR involvement was associated with longer operative times [5]. This suggests that with standard infrarenal EVAR cases at experienced centres, time impact may be minimal. Additionally, since fluoroscopy time, and therefore radiation exposure, was not increased, any additional time is unlikely to have negative implications for patient safety. Overall, the data indicates that DSR involvement in routine, infrarenal EVAR does not compromise case efficiency.

Regarding clinical outcomes, there was no statistically significant correlation between DSR presence and post-operative hospital stay, short- or long-term endoleaks, or mortality. Within this cohort, the lack of clear clinical benefit from DSR involvement suggests that their presence may not be associated with meaningful improvements of patient outcomes. In the context of the limited sample size, the absence of significant differences should be viewed as a reason to question routine DSR involvement rather than being definitive evidence of equivalence.

There exists concerns around the potential influence of DSRs on intraoperative decision-making, including advocating for additional or brand-specific devices. This is comparable to patterns observed in the pharmaceutical industry, where exposure to DSRs was shown to affect physician prescribing behaviours [9]. In this study, there was no statistically significant correlation between DSR presence and rates of endograft use or additional stents. Instead, there was a trend toward lower endograft use in the DSR cohort, but this did not reach significance. Given the limited sample size, these findings should not be used as evidence that DSRs reduce device usage. However, they do challenge the concern of their presence promoting excessive or biased device deployment for monetary profits.

Nonetheless, ethical issues remain important in the discussion of DSR involvement in EVAR. As evidenced by previous work, Canadian vascular surgeons value DSRs for intraoperative guidance [7]. In the current study, at an experienced endovascular centre, the presence of DSRs in over 70% of standard infrarenal cases underscore this perceived value. However, the lack of clear clinical benefit, when weighed against concerns around conflicts of interest and commercial influence, raises important questions. Additionally, DSRs are often present in the OR without explicit patient knowledge of their involvement. The ramifications of this lack of informed consent and transparency have been discussed in other fields [3]. While these ethical considerations were beyond the scope of this study, they highlight the need for future research to use a multidisciplinary approach, with patient and provider perspectives taken into account, in order to better evaluate institutional policies governing industry presence in the OR.

Several limitations affect the interpretation of these findings. The retrospective, single-centre nature introduces risk towards unmeasured confounding variables and the small sample size limits the power to detect modest differences between DSR and control groups with respect to clinical outcomes. The methodological scope also warrants consideration as the patient sample was limited to elective EVAR procedures with standard, infrarenal aneurysm anatomy. This was chosen to ensure uniformity in case complexity and therefore, device selection reflects standard practices in infrarenal EVAR. Nevertheless, details of anatomical complexity and IFU adherence were not considered and should be incorporated in future studies. Furthermore, the study population was situated at a high volume EVAR centre with experienced endovascular surgeons. This limits generalizability as centres with lower case volume or less experience may rely more heavily on DSR and their impact in this environment may differ. These limitations highlight the need for larger, multicentre studies to better understand the context in which DSR involvement is warranted.

## Conclusions

In this study of standard, infrarenal EVAR procedures at an experienced endovascular centre, DSR presence was not associated with statistically significant differences in case efficiency, device utilization or clinical outcomes. Routine DSR participation in this context was not found to have an observable improvement in efficiency or outcomes. Considering the ethical concerns around industry influence and patient privacy, the study calls for thoughtful consideration of when DSR involvement is truly necessary, such as reserving their support for complex cases or in centres with less experienced operators. Large, multicentre studies which captures anatomical complexity, urgency, and IFU adherence, in addition to qualitative patient and physician perspectives, are necessary to better understand the role of DSRs in EVAR cases.

## Supporting information

S1 File Data collection form. (XLSX)
